# Developing a Radiomics Signature for Supratentorial Extra-Ventricular Ependymoma Using Multimodal MR Imaging

**DOI:** 10.3389/fneur.2021.648092

**Published:** 2021-07-22

**Authors:** Apoorva Safai, Sumeet Shinde, Manali Jadhav, Tanay Chougule, Abhilasha Indoria, Manoj Kumar, Vani Santosh, Shumyla Jabeen, Manish Beniwal, Subhash Konar, Jitender Saini, Madhura Ingalhalikar

**Affiliations:** ^1^Symbiosis Center for Medical Image Analysis, Symbiosis Institute of Technology, Symbiosis International University, Pune, India; ^2^Department of Neuroimaging & Interventional Radiology, National Institute of Mental Health & Neurosciences, Bangalore, India; ^3^Department of Neuropathology, National Institute of Mental Health & Neurosciences, Bangalore, India; ^4^Department of Neurosurgery, National Institute of Mental Health & Neurosciences, Bangalore, India

**Keywords:** radiomics, ependymoma, high grade glioblastoma, classification, GBM, glioblastoma multiforme, MRI

## Abstract

**Rationale and Objectives:** To build a machine learning-based diagnostic model that can accurately distinguish adult supratentorial extraventricular ependymoma (STEE) from similarly appearing high-grade gliomas (HGG) using quantitative radiomic signatures from a multi-parametric MRI framework.

**Materials and Methods:** We computed radiomic features on the preprocessed and segmented tumor masks from a pre-operative multimodal MRI dataset [contrast-enhanced T1 (T1ce), T2, fluid-attenuated inversion recovery (FLAIR), apparent diffusion coefficient (ADC)] from STEE (*n* = 15), HGG-Grade IV (HGG-G4) (*n* = 24), and HGG-Grade III (HGG-G3) (*n* = 36) patients, followed by an optimum two-stage feature selection and multiclass classification. Performance of multiple classifiers were evaluated on both unimodal and multimodal feature sets and most discriminative radiomic features involved in classification of STEE from HGG subtypes were obtained.

**Results:** Multimodal features demonstrated higher classification performance over unimodal feature set in discriminating STEE and HGG subtypes with an accuracy of 68% on test data and above 80% on cross validation, along with an overall above 90% specificity. Among unimodal feature sets, those extracted from FLAIR demonstrated high classification performance in delineating all three tumor groups. Texture-based radiomic features particularly from FLAIR were most important in discriminating STEE from HGG-G4, whereas first-order features from T2 and ADC consistently ranked higher in differentiating multiple tumor groups.

**Conclusions:** This study illustrates the utility of radiomics-based multimodal MRI framework in accurately discriminating similarly appearing adult STEE from HGG subtypes. Radiomic features from multiple MRI modalities could capture intricate and complementary information for a robust and highly accurate multiclass tumor classification.

## Introduction

**Figure 1:**
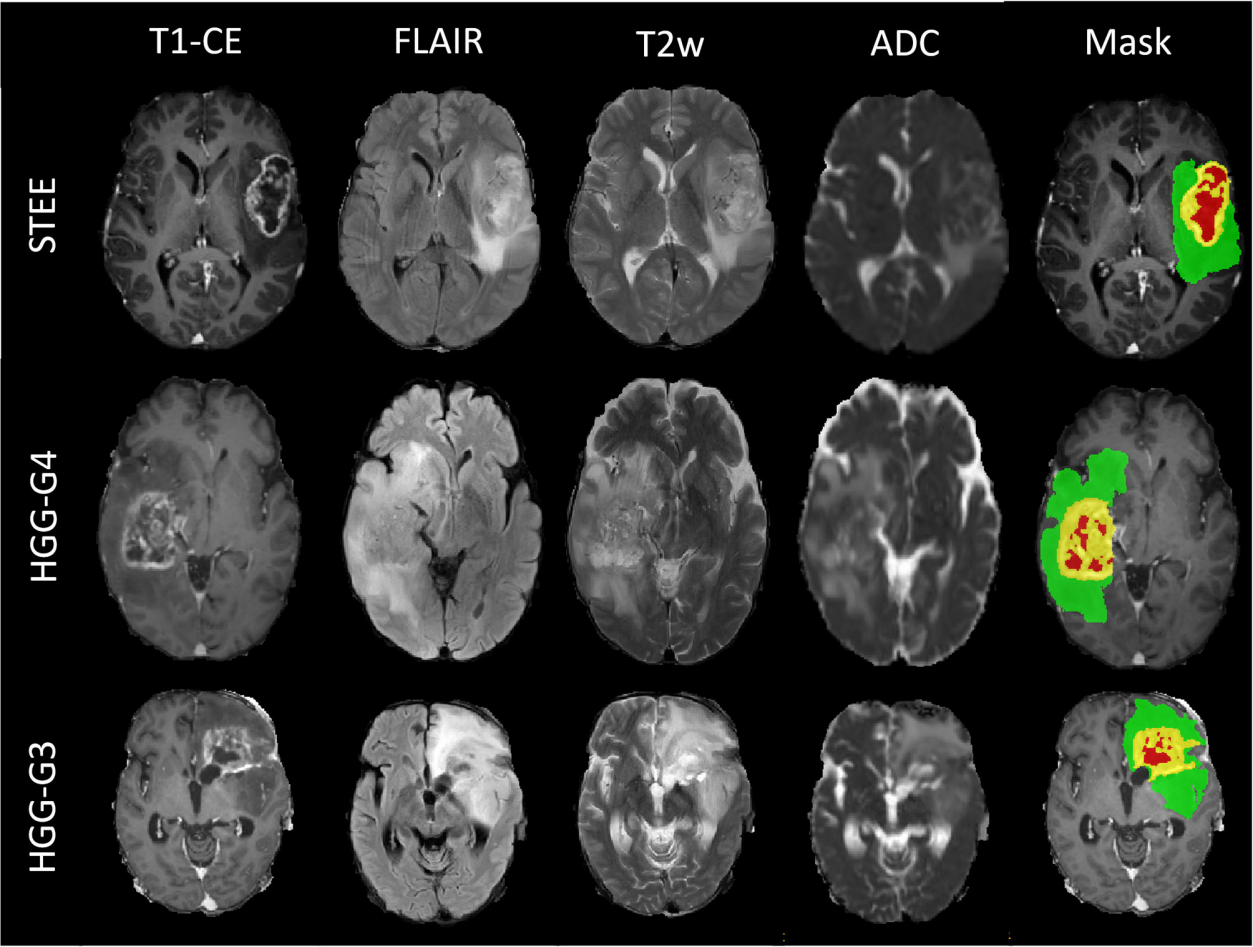
Multimodal magnetic resonance image (MRI) sequences of one supratentorial extraventricular ependymoma (STEE), high-grade gliomas-grade 4 (HGG-G4), and HGG-G3 tumor case with their tumor segmented masks, which shows similarly appearing necrosis tissue (red), diffused enhancement (yellow), and edema tissue (green) in all three tumor groups.

Supratentorial ependymoma are relatively rare neoplasms, which constitute 3–5% of adult intracranial tumors and present with a wide histopathological spectrum (1). Existing literature illustrates that more than 25% of adult ependymoma can be mis-diagnosed, thus, elevating the importance of an accurate diagnosis (2). This is especially true in delineating extra-ventricular supratentorial ependymomas (STEE) from high-grade gliomas (HGG) as the appearance of ependymoma may closely resemble that of a glioblastoma on a magnetic resonance image (MRI) (2–4). STEEs generally appear hypointense on T1-weighted imaging, hyperintense on T2-weighted imaging, with an intermediate to high signal intensity on fluid-attenuated inversion recovery (FLAIR) images and may demonstrate ring- or wreath-like contrast enhancement on gadolinium based T1-weighted imaging as shown in Figure 1 (2, 5–8). Moreover, these lesions demonstrate marked heterogeneity within the tumor. Cystic formation can be noted very frequently, and calcifications are also common that can be seen in ~50% of ependymomas (4, 7, 9). Diffusion-weighted imaging (DWI) from ependymoma demonstrates restricted diffusion within the solid tumor compartment indicating high cellularity of the lesion, while perfusion MRI shows marked increase in cerebral blood volume. Finally, MR spectroscopy demonstrates elevated choline and reduced N-acetyl-aspartate metabolism in tumor lesions (6). The abovementioned features are also observed in HGG-grade III (HGG-G3) referred to as anaplastic astrocytoma and HGG-grade IV (HGG-G4) also known as glioblastoma (3, 10), which are central nervous system neoplasms accounting for 59% of the commonly occurring primary brain tumors (11). Although the pathogenesis and treatment strategy of ependymoma differs significantly from gliomas, and a standard course of management in the case of STEE is not yet established, e.g., chemotherapy and radiotherapy as an adjuvant to resection is a conventional treatment protocol for gliomas (12–14); however, it is not included as part of the accepted standard of care in case of STEE (15). The European Association of Neuro-Oncology guidelines published in 2017 advocate gross total resection followed by adjuvant radiotherapy in grade 3 tumors and adjuvant radiotherapy in low-grade neoplasms if residual tumor is present. Chemotherapy is not advised in adult tumors, although chemotherapy is indicated in children and adults with recurrent tumor in whom primary treatment with resection and radiotherapy has been exhausted (15). It is, therefore, crucial to predict the tumor type to optimize treatment planning and assess the therapeutic interventions, which can subsequently facilitate better outcomes.

Radiomics is an emerging translational field that can extract quantitative features beyond the level of human perception, with an intent to create tumor phenotypic signatures to aid in prognosis, stratification, disease tracking, and treatment response evaluation (16). These features are generally based on geometry (shape), intensity characteristics (histogram), entropy, and numerous image textures that are extracted from the tumoral region. Multivariate classification framework based on these features can facilitate a single probabilistic marker for the tumor type under consideration. The complete analysis aims at delineating tumor types with the ultimate goal of supporting clinical decisions that may consequently improve patient outcomes.

Overall, there is scarce literature regarding neuroimaging findings in ependymomas especially those seen in adult patients. Existing work has demonstrated the utility of a radiomics-based machine learning approach in differential diagnosis of pediatric ependymoma from medulloblastoma and pilocytic astrocytoma on multiple 3D MRI modalities. These studies have reported high classification performance of texture-based features using both conventional T1- and T2-weighted images (17–19) as well as on advanced ADC maps (20, 21) in varying combinations. Recently, studies have also demonstrated the role of radiomics in evaluating treatment response of natural killer cell infusion therapy (22) and novel network-driven approach using proton therapy (23) in pediatric ependymoma. A radiomics-based signature of adult STEE tumors is yet to be established. Apart from ependymomas, radiomics has also shown potential in creating phenotypic signatures of glioma genotypes such as isocitrate dehydrogenase (IDH) (24–26), epidermal growth factor receptor (EGFR) (27), and O^6^-methylguanine-DNA-methyl-transferase (MGMT) (28–31). In glioblastoma, multimodal MRI-based quantitative radiomic features have shown to predict tumor recurrence (32) with better performance than traditional qualitative approaches such as visualization of contrast-enhanced MRI and perfusion kinematic changes in discriminating recurrence from radiation necrosis (33, 34). These studies provide outcomes that evidently encourage the use of radiomics-based multimodal MRI in combination with machine learning framework to create an imaging marker of adult STEE tumor, which can accurately delineate it from HGG tumors.

This study aims to characterize adult STEEs using an underlying phenotypic radiomics-based signature from multimodal MRI images, which not only predicts but also portrays the textural patterns that mark the uniqueness of these tumors on MRI and discriminate them from HGG, thereby serving as a potential biomarker. Such a non-invasive differential prognostic signature of STEE can aid in better diagnosis, improve clinical decision making, and timely therapeutic intervention with better outcomes.

## Materials and Methods

### Study Cohort and Imaging

Our dataset consisted of a clinical cohort of 75 adult tumor patients that included 15 patients (age = 27.2 ± 11.73 years, M:F = 8:7) with grade 2 and grade 3 STEEs, 36 patients with HGG-G3 (age = 39.30 ± 11.64 years, M:F = 22:14), and 24 HGG-G4 patients (age = 48.8 ± 15.76 years, M:F = 11:13). All patients included in this study had undergone surgical resection and standard post-surgical care and were identified retrospectively after reviewing the medical records. Final diagnosis was confirmed based on the histopathological examination of the resected tissue. Out of the complete cohort, 77% scanned were performed on a Philips Achieva 3.0 T MRI scanner, while others were scanned on a 3.0 T Siemens Skyra MRI system. Multiple sequences were acquired as standard clinical MRI; however, we restricted our analysis to gadolinium-enhanced T1-weighted (T1ce), fluid attenuation inversion recovery (FLAIR), T2-weighted imaging, and apparent diffusion coefficient (ADC) maps. T1ce scans were obtained using TR/TE = 8.0/3.7 ms using TFE sequence on Philips scanner, while TR/TE = 1,800–2,200/2.3–2.6 ms using T1 MPRAGE sequence on Siemens with 1 × 1 × 1 mm isotropic resolution. T2-weighted imaging protocol consisted of TR/TE ranging from 3,600 to 6,000/80 to 99 ms and 0.5 × 0.5 mm resolution in the axial plane. FLAIR images were acquired using TR/TE/TI of 11,000/125/2,800 within the plane resolution of 0.5 × 0.5 mm. ADC maps were acquired using the DWI sequence. The institute review ethics committee approved the study and the informed consent of the patient was waived off as it was a retrospective study.

### Image Processing and Radiomics

**Figure 2:**
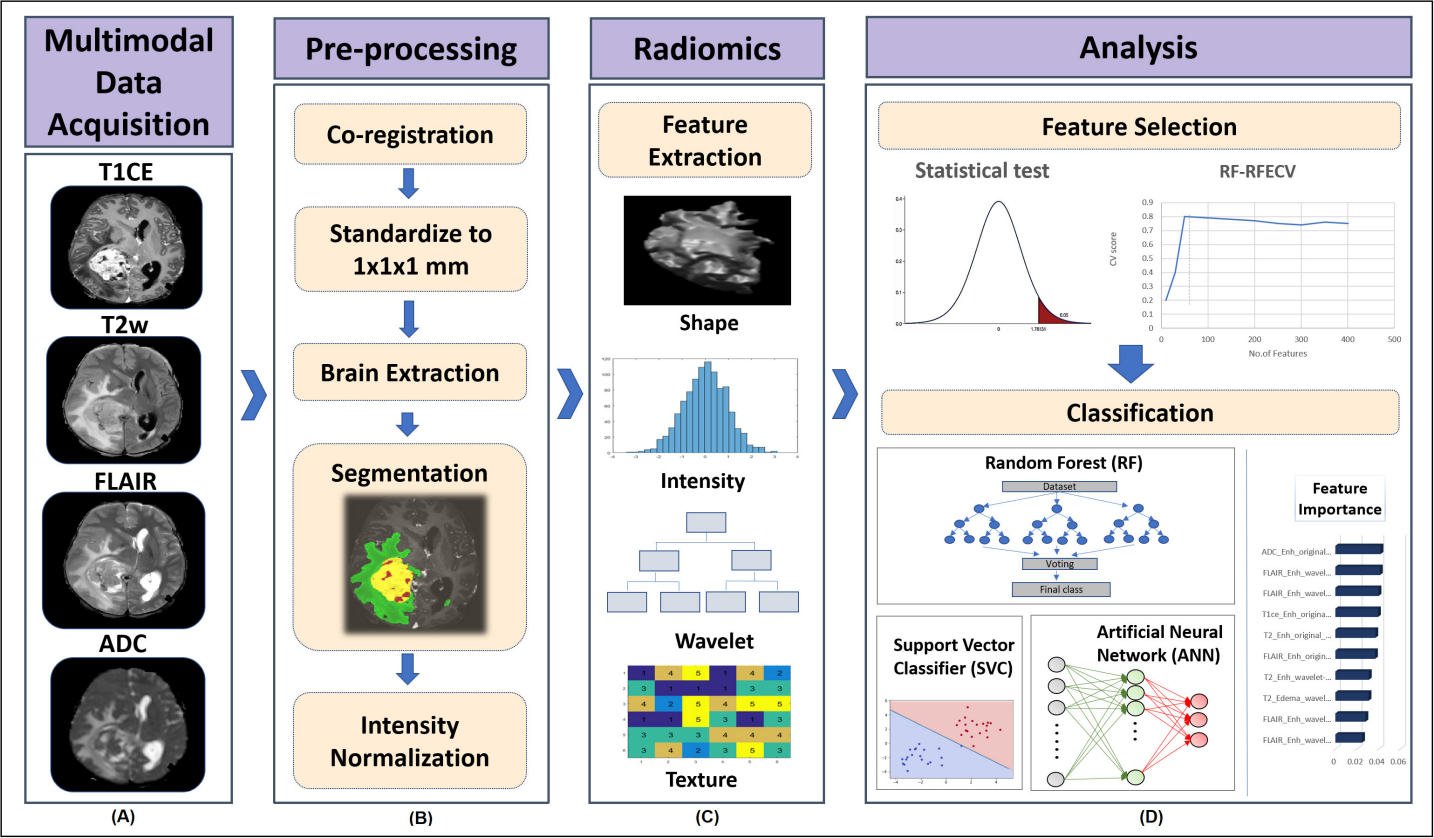
Processing pipeline implemented for classification of tumor subgroups includes **(A)** Acquisition of multiple MRI modalities such as gadolinium enhanced T1-weighted (T1ce), fluid attenuation inversion recovery (FLAIR), T2-weighted imaging, and apparent diffusion coefficient (ADC) maps. **(B)** Pre-processing of MRI scans to segment different tumor tissues involved co-registration of FLAIR, T2, and ADC scans to T1ce scan and standardizing all modalities to 1 mm iso voxel size using advanced normalization tool (ANTs) software, brain extraction using all modalities using FSL's BET, segmentation of enhancing tumor, edema, and necrosis using deep learning (DeepMedic) model and its manual correction followed by intensity normalization. **(C)** Radiomic features such as shape, intensity-based histograms, and texture features were computed from original segmented mask of each tumor tissue using PyRadiomics. **(D)** Dual stage feature selection was performed on normalized radiomic features of each modality, where the first stage involved extraction of significantly varying features between tumor groups using ANOVA F-test with a *p*-value cut-off of 1 xe^−06^. The second stage of feature selection was performed on features selected from stage 1 by implementing Random Forest based Recursive Feature Elimination with Cross Validation (RF-RFECV), such that initially a RF model was fit in a 5 fold-CV framework, with elimination of features at each cross validation (CV) based on their feature importance score and the model was recursively retrained on updated feature set until a minimum of 10 features were obtained that gave high accuracy across all the CVs. Classification performance of selected multimodal radiomic features in distinguishing different tumor groups was assessed by implementing a multiclass classification modal using RF, support vector classifier (SVC) and artificial neural network (ANN) classifiers. SVC coefficients were used to obtain the most important features.

The detailed pre-processing of MRI images and radiomics pipeline implemented in this study is shown in Figure 2. Initially, for all the subjects, FLAIR, T2, and ADC maps were resampled to 1.0 mm iso-voxel and registered to T1ce image using a 6 degree of freedom rigid body transformation. T1ce images were further registered to standard MNI-spaced image using affine transformation, and this transformation matrix was further applied to FLAIR, T2, and ADC maps to have them all in a uniform sampling space with a common origin. All registration steps were performed using advanced normalization tool (ANTs) toolbox (35). Brain extraction for all co-registered modalities was performed using FSL's BET (36), followed by segmentation of enhancing tumor, edema, and necrosis using a deep learning model (DeepMedic) (37). This multiscale 3D convolutional neural network (CNN) model was trained on multimodal images (T1ce, T2, and FLAIR images) and labels from BRATS-2018 (https://www.med.upenn.edu/sbia/brats2018/data.html) data (training *n* = 206, validation *n* = 52). The segmented output masks were corrected manually by an expert annotator (M.J), followed by intensity normalization and computation of radiomic features using PyRadiomics 2.2.0 library (38). The feature set used included 3D shape-based features, statistical features, gray-level co-occurrence matrix (GLCM), gray-level dependent matrix (GLDM), gray-level run length matrix (GLRLM), gray-level size zone matrix (GLSZM), and neighboring gray tone difference matrix (NGTDM). These features were also computed on filtered images where the filters used were Laplacian, wavelets, Gaussian, curvature flow, box mean, and box sigma. For each subject, a total of 11,274 features were computed, comprising 1,409 features from each of the four modalities, making a total of 5,637 features for each of the two tumor masks (edema and T1ce-based tumor tissue enhancing). We excluded the third region of interest (necrosis/cyst) from further analysis due to its heterogeneous tissue composition, which involved parts of non-enhancing tumor, cysts in some cases, and tumor necrosis.

### Feature Selection

**Figure 3:**
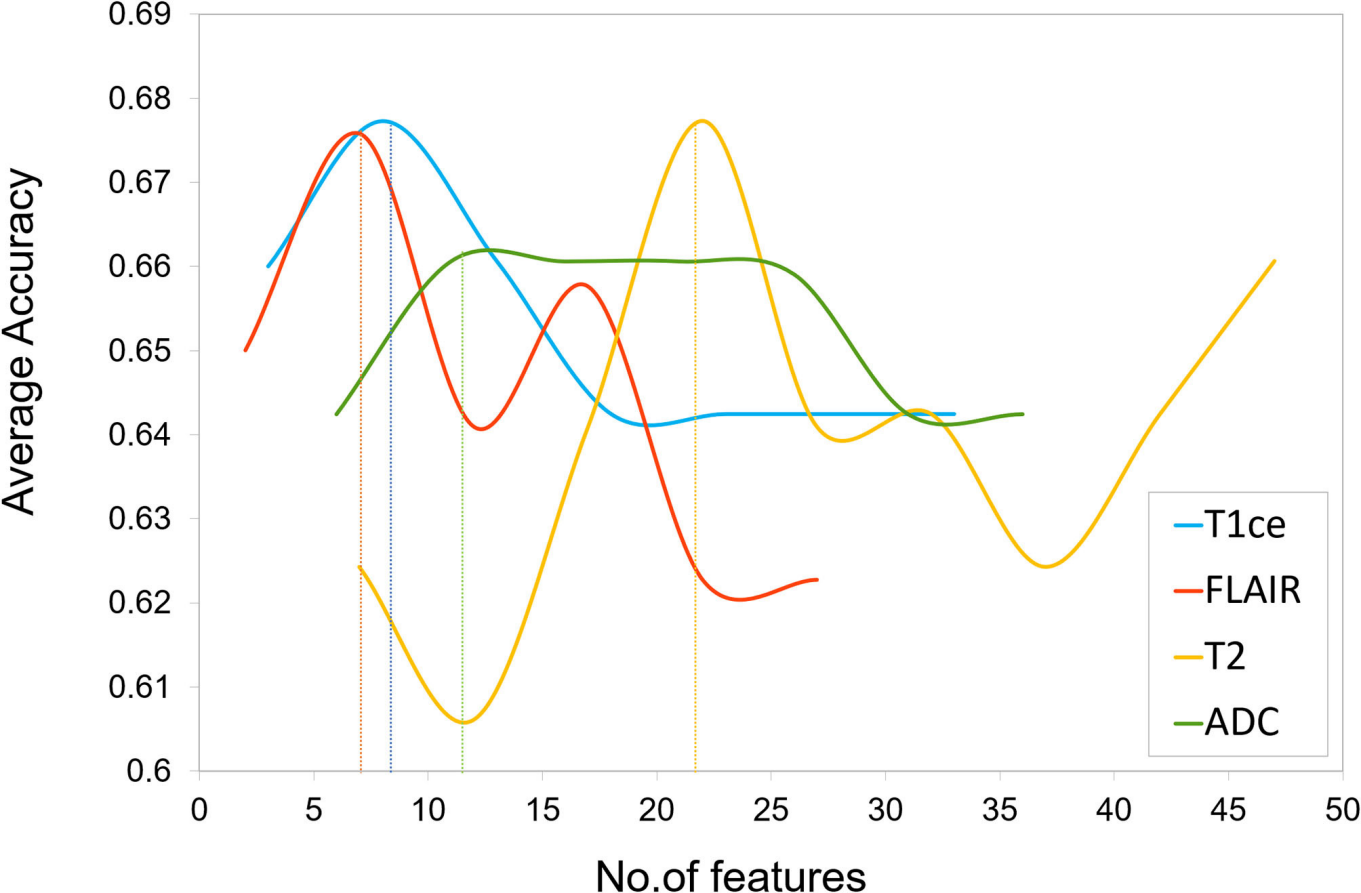
Feature selection plot of number of features vs. average cross validation (CV) accuracy for each unimodal MRI feature set obtained by implementing Random Forest based Recursive Feature Elimination with Cross Validation (RF-RFECV) on training data. The vertical dotted line indicates a threshold on number of features below which a drop in average accuracy was observed.

Multivariate classifiers may overfit on the input radiomic features, due to their enormously large size compared with the available sample size. To alleviate this issue, feature selection was performed to obtain an optimum feature set by removing redundant features and reducing feature dimensionality of the computed radiomic features. First, a 75–25% train-test split was applied on the dataset, with 56 subjects in the training set and 19 subjects in the test set. We then normalized all radiomic features using min–max normalization and implemented a two-stage feature selection strategy on the training dataset. In the first stage, features from each modality were statistically compared between groups using an ANOVA F-test. A *p*-value cutoff of 1 × e^−06^ was applied per modality to obtain up to 50 significant radiomic features per modality. The second stage of feature selection was performed on the selected features obtained from stage 1 by implementing random forest-based recursive feature elimination with cross validation (RF-RFECV) (39–41). This two-stage feature selection process was applied individually on all four modalities. RF-RFECV was implemented by first fitting a random forest (RF) model (42) in a cross-validation (CV) framework on the training data using a fivefold CV. The least important features were eliminated (minimum step size-5) after every CV based on their feature importance scores pertaining to that CV, and an averaged fivefold CV accuracy was noted at every elimination. In the next step, the model was recursively retrained on updated feature set until a minimum of 10 features were obtained that gave high accuracy across all the CVs as shown in Figure 3. Thus, for each modality, a specific number of important selected radiomic features were used as input in the classification models.

### Classification Models

To distinguish between STEE, HGG-G3, and HGG-G4 tumor types, we performed multiclass classification using the radiomic feature set of each modality individually in a unimodal setup, as well as by combining features from all modalities in a multimodal setup. Additionally, we employed multiple machine learning classification algorithms such as random forest (RF), support vector classifier (SVC), and artificial neural network (ANN) on both unimodal and multimodal feature sets to evaluate classification performance using different models. Random forests are decision tree-based ensemble learning classification algorithms that involve fitting multiple decision tree classifiers on random subsamples of the data and predicting the final class by aggregating votes or predictions from different decision trees. RF algorithms control overfitting of the model that may occur in a single decision tree (42). SVC is a supervised learning algorithm that performs linear or non-linear classification by transforming data to a higher dimensional space using a kernel function and constructing an optimal hyperplane. The hyperplane classifies data points into different classes by maximizing the distance between the nearest point on its either side (43). ANN is a biologically inspired feed forward neural networks that consists of an input layer, hidden layer, and output layer with nodes that act as activation functions and a back-propagation algorithm that trains the model for classification (44). We implemented RF classification model using 10,000 trees, maximum depth = 2 and a maximum of square root of input features per tree with “Gini” criterion as the loss. SVC was implemented using a linear kernel on the training dataset having a c value of 1. A three-layer ANN was implemented with a single hidden layer of five hidden units followed by a softmax activation function at the output layer. The model was optimized using Adam optimizer, with a learning rate = 0.005 and binary cross entropy as the loss function. All three classification models were trained on a training set of 56 subjects and tested on 19 subjects, in both unimodal as well as in multimodal setup. Additionally, a leave-one-out-type cross validation (LOOCV) was implemented on the training set for each classifier to validate the model. To avoid classification bias in favor of majority class due to our unbalanced group samples and to attain robust prediction, we augmented the training data using a borderline Synthetic Minority Oversampling Technique (SMOTE) (45). Classification performance of the three classifier models were compared by evaluating their performance metrices, which included macro-averaged accuracy, F1-score, and AUROC (area under receiver operating curve), whereas the class-specific classification performance was evaluated using precision, recall, F1-score, and AUROC. To determine the most discriminative features involved in classification, we further obtained SVC coefficients for each pair of classes for unimodal as well as multimodal feature sets, with a high coefficient score implying high contribution of the feature in the classification of tumor groups.

## Results

### Clinical and Radiological Characteristics of Tumors

Qualitative MRI features of patients with STEE, HGG-G3, and HGG-G4 are depicted in Figure 1. It demonstrates the similarity in the imaging findings of STEE and HGG with common occurrences of edema, heterogenous enhancement, necrosis, hemorrhage, and diffusion restriction in all three tumor groups. Detailed radiological findings for STEEs are reported in (4).

### Feature Selection and Classification of Unimodal Radiomic Features

The two-stage feature selection process on each MRI modality generated an optimum final feature set per modality (T1ce-10 features, FLAIR-10 features, T2-22 features, ADC-11 features) based on the maximum average CV accuracy obtained using RF-RFECV, as shown in Figure 3. The performance of classification models using each unimodal feature set is mentioned in Table 1. Among all modalities, radiomic features obtained from FLAIR showed highest test accuracy of more than 60% using RF and SVC classifiers, respectively. ANN showed a test accuracy of 57%, a maximum CV accuracy of 75%, and AUROC of 85% among the three classifiers. More than 75% AUROC were observed consistently across all classifiers, along with high F1-score and sensitivity using FLAIR modality. High specificity was observed across all models for all modalities. As SVC model provided balanced performance throughout all modalities, we further assessed classification performance for each tumor group in a one vs. all manner using the SVC model as shown in Table 2A. Overall, all modalities gave high performance in identifying the HGG-G3 group, while FLAIR modality showed high precision in distinguishing all three tumor groups. In accordance with SVC results, RF and ANN classifiers also showed high performance on FLAIR and additionally on ADC and T2 modalities, respectively, as shown in Supplementary Table 1.

**Table 1:** Classification results from unimodal and multimodal feature sets (testing/cross) validation (CV).

**Classifier**	**Accuracy**	**Sensitivity**	**Specificity**	**F1-score**	**AUROC**
**A) Classification based on T1ce feature set**					
RF	0.52/0.69	0.00/0.00	0.93/0.93	0.39/0.52	0.70/0.70
SVC	0.47/0.62	0.50/0.36	0.73/0.77	0.36/0.54	0.75/0.76
ANN	0.52/0.71	0.00/0.18	1.00/0.91	0.38/0.60	0.54/0.80
**B) Classification based on FLAIR feature set**					
RF	**0.63/0.53**	0.5/0.18	0.93/0.73	0.61/0.44	**0.76/0.73**
SVC	**0.63/0.67**	0.5/0.18	0.93/0.91	0.61/0.57	**0.79/0.80**
ANN	**0.57/0.75**	0.50/0.36	0.86/0.93	0.53/0.68	**0.75/0.85**
**C) Classification based on T2 feature set**					
RF	0.42/0.67	0.00/0.27	0.73/0.84	0.30/0.59	0.67/0.79
SVC	0.47/0.58	0.25/0.18	0.86/0.75	0.40/0.48	0.75/0.78
ANN	0.73/0.67	1.00/0.54	0.86/0.75	0.72/0.60	0.78/0.83
**D) Classification based on ADC feature set**					
RF	0.57/0.67	0.25/0.27	0.93/0.88	0.51/0.58	0.78/0.78
SVC	0.42/0.64	0.25/0.27	0.73/0.82	0.31/0.55	0.75/0.82
ANN	0.52/0.69	0.00/0.27	0.93/0.86	0.39/0.60	0.64/0.80
**E) Classification based on multimodal feature set**					
RF	0.53/0.75	0.50/0.36	0.80/0.95	0.49/0.73	0.77/0.80
SVC	**0.68/0.80**	**0.75/0.45**	**0.93/0.95**	**0.68/0.74**	**0.78/0.84**
ANN	**0.68/0.87**	**0.75/0.81**	**0.93/0.91**	**0.68/0.85**	**0.78/0.94**

*Accuracy and f1-score (macro averaged) are overall classification scores, whereas sensitivity and specificity are for STEE class*.

*AUROC, area under receiver operating curve; RF, random forest; SVC, Support Vector Classifier; ANN, artificial neural network; FLAIR, Fluid Attenuated Inversion Recovery; ADC, apparent diffusion coefficient; STEE, Supratentorial Extraventricular Ependymoma*.

*Highlighted scores indicate high classification performance*.

**Table 2:** Class-specific performance metrices of classifiers on unimodal and multimodal feature sets.

**Classifier**	**Class**	**Precision**	**Recall**	**F1-score**
**A) Class-specific performance metrices of SVC on unimodal feature set**				
T1ce	STEE	0.33/0.29	0.50/0.36	0.40/0.32
	HGG-G4	0.00/0.54	0.00/0.39	0.00/0.45
	HGG-G3	0.58/0.83	0.78/0.89	0.67/0.86
FLAIR	STEE	**0.67/0.33**	**0.50/0.18**	**0.57/0.24**
	HGG-G4	**0.75/0.57**	**0.50/0.67**	**0.60/0.62**
	HGG-G3	**0.58/0.83**	**0.78/0.89**	**0.67/0.86**
T2	STEE	0.33/0.15	0.25/0.18	0.29/0.17
	HGG-G4	0.17/0.40	0.17/0.33	0.17/0.36
	HGG-G3	0.70/0.89	0.78/0.93	0.74/0.91
ADC	STEE	0.20/0.27	0.25/0.27	0.22/0.27
	HGG-G4	0.00/0.56	0.00/0.50	0.00/0.53
	HGG-G3	0.64/0.83	0.78/0.89	0.70/0.86
**B) Class-specific performance metrices on multimodal feature set**				
RF	STEE	0.50/0.38	0.25/0.27	0.33/0.32
	HGG-G4	0.50/0.58	0.50/0.61	0.50/0.59
	HGG-G3	0.64/0.83	0.78/0.89	0.70/0.86
SVC	STEE	**0.75/0.71**	**0.75/0.45**	**0.75/0.56**
	HGG-G4	**0.60/0.70**	**0.50/0.78**	**0.55/0.74**
	HGG-G3	**0.70/0.90**	**0.78/0.96**	**0.74/0.93**
ANN	STEE	**0.75/0.69**	**0.75/0.82**	**0.75/0.75**
	HGG-G4	**0.50/0.88**	**0.50/0.83**	**0.50/0.86**
	HGG-G3	**0.78/0.96**	**0.78/0.93**	**0.78/0.94**

*HGG, high-grade gliomas. Highlighted scores indicate high classification performance*.

### Classification Performance of Multimodal Radiomic Features

**Figure 4:**
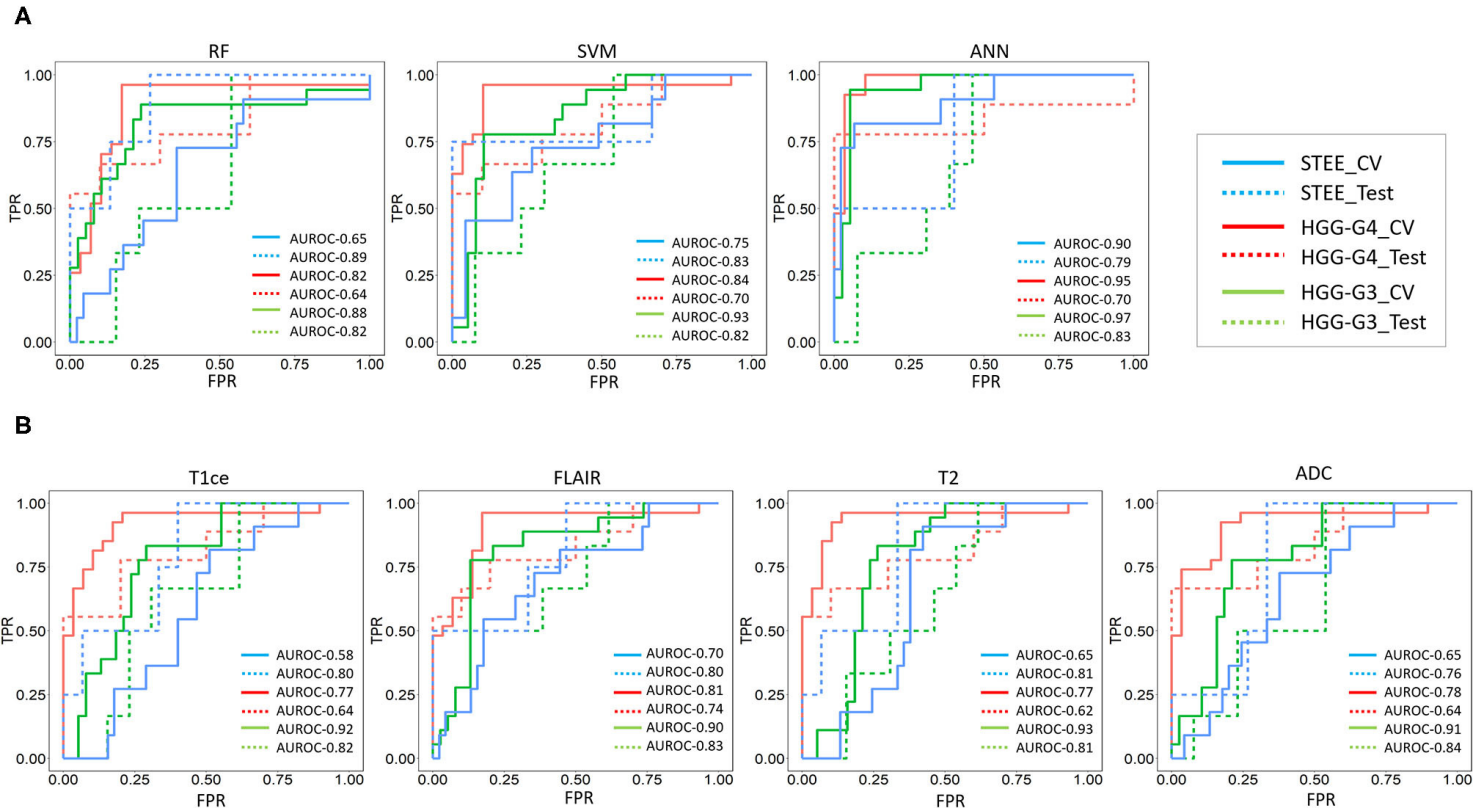
**(A)** Receiver operating characteristic (ROC) curves for classification on multimodal feature set- ROC curves for multimodal features-based classification of multiple tumor groups (STEE: supratentorial extraventricular ependymoma, HGG-G4: high-grade glioma-grade 4, HGG-G3: high-grade glioma-grade3) using random forest (RF), support vector classifier (SVC), and artificial neural network (ANN) classifiers based on CV and test data. **(B)** ROC curves for SVC on unimodal features set-ROC curves unimodal [gadolinium enhanced T1-weighted (T1ce), fluid attenuation inversion recovery (FLAIR), T2-weighted imaging, and apparent diffusion coefficient (ADC)] feature-based SVC classification of multiple tumor groups using CV and test data.

Classification performance of RF, SVC, and ANN models on multimodal feature set is provided in Table 1E. Both SVC and ANN showed 68% accuracy, 75% sensitivity, and 93% specificity on test data, while on CV, both classifiers demonstrated more than 80% accuracy and 90% specificity, along with 81% sensitivity using the ANN classifier. All classifiers showed an AUROC of 78% or more on test and CV; a maximum of 94% was attained on CV of ANN as shown in Table 1D and Figure 4, respectively. Higher accuracy and AUROC were obtained on multimodal feature set compared with unimodal feature sets, across all classifiers. Similar to the unimodal feature set, all classifiers showed high performance in identifying the HGG-G3 group on multimodal feature set as shown in Table 2B.

Feature importances evaluated on multimodal feature set using SVC are shown in Table 3. Radiomic features of FLAIR on enhancement tissue, T2, and ADC on edema tissue were the commonly occurring top 10 features that were most discriminative between any two tumor groups. Particularly, the texture-based GLRLM (gray-level run length matrix) feature–run length non-uniformity (RLN) was among the topmost frequently occurring important feature in distinguishing STEE from HGG-G4 as reported in Table 3A. From T2 and ADC modality, first-order features and texture-based NGTDM (neighboring gray tone difference matrix)-busyness and GLDM (gray-level dependence matrix)–high gray-level emphasis (HGLE) features on edema tissue were important in classifying STEE from HGG-G3. The first-order features and GLRLM features were important in distinguishing the HGG-G4 from the HGG-G3 tumor group. Interestingly, the GLDM feature from T2 edema was significantly distinct between the STEE and HGG-G4 tumor groups and was also found to be the most important feature in classifying HGG-G3 from HGG-G4 and STEE, respectively. Overall, the first-order features from T2 and ADC on edema tissue were highlighted as the commonly seen important features in discriminating STEE and HGG tumor subtypes, whereas FLAIR-based GLRLM texture feature on enhancement was particularly important in classifying STEE from HGG-G4.

**Table 3:** Feature importances obtained using SVC coefficient scores on multimodal feature set.

**Sr.no**.	**Features**	**Coefficients**	***p*-value**	***t*-stat**
**A) STEE vs. HGG-G4**				
1	ADC_Edema_wavelet-LHL_firstorder_InterquartileRange	1.214	0.554	**0.598**
2	FLAIR_Enh_wavelet-LHH_glrlm_RunLengthNonUniformity	1.114	0.591	0.546
3	FLAIR_Enh_wavelet-HLL_glrlm_RunLengthNonUniformity	0.987	0.831	0.216
4	FLAIR_Enh_wavelet-LHL_glrlm_RunLengthNonUniformity	0.967	0.790	0.269
5	T2_Edema_wavelet-HHL_gldm_HighGrayLevelEmphasis	0.790	0.055	−1.983
6	ADC_Enh_original_shape_LeastAxisLength	0.768	0.233	−1.225
7	T2_Edema_gradient_firstorder_Mean	0.759	0.586	0.550
8	FLAIR_Enh_wavelet-HHH_glrlm_GrayLevelNonUniformity	0.620	0.602	0.530
9	T2_Edema_gradient_firstorder_Median	0.579	0.497	0.687
10	T1ce_Enh_wavelet-HLH_ngtdm_Busyness	0.564	0.960	−0.051
**B) STEE vs. HGG-G3**				
1	T2_Edema_wavelet-HHL_gldm_HighGrayLevelEmphasis	1.145	0.000	−7.229
2	T2_Edema_gradient_firstorder_Median	0.949	0.000	7.181
3	ADC_Edema_wavelet-LHL_firstorder_InterquartileRange	0.762	0.000	5.028
4	T2_Edema_wavelet-HHH_glrlm_RunEntropy	0.598	0.000	−5.882
5	ADC_Edema_gradient_firstorder_Mean	0.582	0.000	5.493
6	T2_Enh_wavelet-LHL_ngtdm_Busyness	0.414	0.005	3.241
7	T2_Enh_wavelet-LHH_ngtdm_Busyness	0.378	0.004	3.254
8	ADC_Edema_gradient_firstorder_Median	0.360	0.000	5.111
9	T2_Edema_gradient_firstorder_Mean	0.351	0.006	3.160
10	T2_Enh_wavelet-HLH_ngtdm_Busyness	0.347	0.000	5.729
**C) HGG-G4 vs. HGG-G3**				
1	T2_Edema_wavelet-HHL_gldm_HighGrayLevelEmphasis	0.721	0.000	−5.617
2	ADC_Edema_gradient_firstorder_Median	0.647	0.000	6.007
3	ADC_Edema_gradient_firstorder_Mean	0.586	0.000	5.902
4	T2_Edema_gradient_firstorder_Median	0.532	0.000	6.415
5	ADC_Edema_wavelet-LHL_firstorder_InterquartileRange	0.500	0.000	5.134
6	T2_Edema_square_glrlm_RunVariance	0.419	0.000	−5.658
7	T2_Edema_lbp-2D_glrlm_RunVariance	0.419	0.000	−5.658
8	T2_Edema_gradient_glrlm_RunVariance	0.419	0.000	−5.658
9	T2_Edema_exponential_glrlm_RunVariance	0.419	0.000	−5.658
10	T1ce_Edema_square_glrlm_RunVariance	0.419	0.000	−5.658

*Highlighted scores indicate high classification performance*.

## Discussion

Radiological manifestations of STEEs are complicated and can often be mis-diagnosed as HGGs, which are frequently occurring neoplasms of the brain. This study aims at identifying multimodal imaging signatures of STEE tumors through a detailed radiomics-based quantitative evaluation. Our results demonstrate that coalescence of multiple MRI modalities leads to a superior classification performance compared with a single modality. FLAIR, T2, and ADC emerged as the highly discriminative modalities, whereas texture and higher-order statistical features were able to capture intricate imaging markers that could aid in accurately predicting STEE from HGG tumors.

Differential diagnosis of STEE is more challenging compared with infratentorial ependymomas as the latter mostly manifests in the ventricles, whereas STEE may appear outside the ventricles, in cortical regions, similar to other high-grade tumors such as anaplastic astrocytoma and glioblastoma (2, 5). Necrosis, internal hemorrhages, tissue heterogeneity, ring enhancement, significant edema, choline/N-acetylasepartate metabolic ratio on MR spectroscopy, occurrence of tumor in brain parenchyma, and infiltration into the contralateral frontal hemisphere (2, 5–7, 9) are some of the common characteristics of these tumors. Radiographic imaging markers of STEE tumors manifest on conventional MRI sequences as a heterogenous signal intensity on T1- and T2-weighted images and as variable appearance on FLAIR images (2, 5–8). Cyst and calcification are commonly occurring attributes of STEE tumors (4, 7, 9). Higher ADC values close to the white matter are also observed in these tumors (4). DWI and perfusion imaging illustrate restricted diffusion and high relative cerebral blood volume values, respectively, in STEEs, which are similar to high-grade anaplastic astrocytoma and glioblastoma (46). However, till date, there is no consensus on an established differential diagnosis of STEE, primarily due to its rare occurrence, which makes it difficult to conduct research studies and develop a validated biomarker. This often results in mis-diagnosis and poor prognosis of STEEs.

We investigated multiclass classification performance of unimodal as well as multimodal feature sets using RF, SVC, and ANN classifiers. Both SVC and ANN classifiers illustrated high classification performance. Multimodal features demonstrated consistently high AUROC of more than 75% and high CV accuracy of more than 80% from these two classifiers for each tumor type, as shown in Table 1E and Figure 4, suggesting that a multiparametric MRI framework would be more efficient and robust in classifying STEE from HGGs compared with a single modality. Among the unimodal feature sets, radiomic features computed from FLAIR modality provided maximum macro average classification accuracy of more than 55% as shown in Table 1B, which is significantly higher compared with the baseline 33% accuracy for multiclass classification. Moreover, in comparison with other modalities, FLAIR features demonstrated consistently high AUROC for all tumor types while assessing class-specific comparison of SVC as shown in Figure 4 and Table 2B. T2 and ADC modalities performed better in delineating HGG-G3 tumors from other tumor groups as shown in Table 2B.

For a comprehensive understanding of each modality's contribution in classification of different tumor types in a multiparametric framework, we ranked the most important features based on the SVC coefficient score obtained from multimodal feature sets as shown in Table 3. Overall, first-order features such as mean, median, and interquartile range of edema tissue from T2 and ADC along with GLDM-HGLE feature from T2 were the commonly occurring most discriminative features in classifying different tumor groups. The GLDM-HGLE feature implies a larger concentration of high gray-level intensities. It was most significant (*p* = 0.05) in classifying STEE from HGG-G4 and was found to be lower in STEE compared with the HGG groups and higher in HGG-G3 compared with HGG-G4. Among other texture features, the GLRLM feature indicative of homogeneity within the particular tissue types was the commonly occurring important feature in the classification of STEE from HGG-G4. STEE was found to be more homogenous than HGG-G4 on enhancement tissue of FLAIR. NGTDM-busyness was important in classifying STEE from HGG-G3 and was higher in STEE on edema tissue of T2 modality. The busyness feature indicates a rapid change in intensities within a pixel and its neighborhood. Thus, texture-based features signifying concentration of high intensities, homogeneity, and intensity fluctuations between pixel neighborhoods were some of the key radiomics attributes that could distinguish different tumor types.

There were a few limitations to this study. The sample size of different tumor groups was unbalanced. Owing to the extremely rare occurrence of STEEs, acquiring a large number of MRI scans of these tumors is difficult. However, we controlled for this limitation by implementing data augmentation using the SMOTE technique, which provided a balanced dataset for our multiclass classification. Another limitation was that multiscanner data were used in the analysis. To control for scanner differences, we normalized the intensity of the data during pre-processing stage, prior to classification.

In conclusion, this study develops a potential quantitative radiomics signature for accurately differentiating STEE from HGGs using multimodal MRI. Radiomics features from FLAIR modality can aid predominantly in the classification of STEE and HGG-G4 tumor, whereas a multiparametric radiomics approach constituting particularly of FLAIR, ADC, and T2 modalities could provide intricate and complementary information that could aid in highly accurate classification of STEE and HGGs tumors. This work, thus, emphasizes the utility of radiomics-based multimodal MRI framework in pre-operative clinical diagnosis and effective treatment planning.

## Data Availability Statement

The raw data supporting the conclusions of this article will be made available by the authors. There are ethical restrictions on sharing of clinical and imaging data as it contains sensitive patient information. Data access will be provided upon the approval of the Institutional Ethics Committee.

## Ethics Statement

The studies involving human participants were reviewed and approved by Institute Ethics Committee NIMHANS and patient informed consent was waived off due to retrospective study.

## Author's Note

Adult Supratentorial Extraventricular Ependymoma (STEE) are rare neoplasms that are often misdiagnosed as high-grade gliomas (HGG) due to their similar radiological manifestation on MRI. However, the pathogenesis and treatment plan of ependymoma differs significantly from gliomas, and hence an early and accurate diagnosis is crucial. Our proposed machine learning based diagnostic model can accurately distinguish adult STEE from HGG subtypes using quantitative radiomic signatures from a multi-model MRI data. Quantitative radiomic markers such as texture and first order statistics from multimodal MRI can capture intricate and complementary information and thus aid in a robust multiclass tumor classification of STEE and HGG subtypes.

## Author Contributions

AS, SS, MJ, TC, and AI: data analysis and processing. MK, JS, and MI: manuscript writing and editing. VS, SJ, MB, and SK: clinical evaluation.

## Conflict of Interest

The authors declare that the research was conducted in the absence of any commercial or financial relationships that could be construed as a potential conflict of interest.
