# Supplementary material for: Developing a Radiomics Signature for Supratentorial Extra-Ventricular Ependymoma Using Multimodal MR Imaging
Source: Front Neurol. 2021 Jul 22;12:648092. doi: 10.3389/fneur.2021.648092 (PMC8339322; doi:10.3389/fneur.2021.648092)
Supplement: Supplementary file 1 [file Table_1.DOCX]

Supplementary Material

Table S1: Class specific performance metrices of RF and ANN classifiers on unimodal feature sets.

| 1. Class specific performance metrices of RF classifier on unimodal feature set | | | | |
| --- | --- | --- | --- | --- |
| Classifier | Class | Precision | Recall | F1-score |
| T1ce | STEE | 0.00/0.00 | 0.00/0.00 | 0.00/0.00 |
|  | HGG-G4 | 0.50/0.61 | 0.50/0.78 | 0.50/0.68 |
|  | HGG-G3 | 0.58/0.83 | 0.78/0.93 | 0.67/0.88 |
|  |  |  |  |  |
| FLAIR | STEE | 0.67/0.14 | 0.50/0.18 | 0.57/0.16 |
|  | HGG-G4 | 0.75/0.36 | 0.50/0.28 | 0.60/0.31 |
|  | HGG-G3 | 0.58/0.82 | 0.78/0.85 | 0.67/0.84 |
|  |  |  |  |  |
| T2 | STEE | 0.00/0.30 | 0.00/0.27 | 0.00/0.29 |
|  | HGG-G4 | 0.25/0.65 | 0.17/0.61 | 0.20/0.63 |
|  | HGG-G3 | 0.64/0.83 | 0.78/0.89 | 0.70/0.86 |
|  |  |  |  |  |
| ADC | STEE | 0.50/0.20 | 0.25/0.18 | 0.33/0.19 |
|  | HGG-G4 | 0.50/0.59 | 0.50/0.56 | 0.50/0.57 |
|  | HGG-G3 | 0.64/0.83 | 0.78/0.89 | 0.70/0.86 |
|  |  |  |  |  |
| 1. Class specific performance metrices of ANN classifier on unimodal feature set | | | | |
| Classifier | Class | Precision | Recall | F1-score |
| T1ce | STEE | 0.00/0.33 | 0.00/0.18 | 0.00/0.24 |
|  | HGG-G4 | 0.43/0.65 | 0.50/0.72 | 0.46/0.68 |
|  | HGG-G3 | 0.58/0.83 | 0.78/0.93 | 0.67/0.88 |
|  |  |  |  |  |
| FLAIR | STEE | 0.50/0.57 | 0.50/0.36 | 0.50/0.44 |
|  | HGG-G4 | 0.67/0.70 | 0.33/0.78 | 0.44/0.74 |
|  | HGG-G3 | 0.58/0.83 | 0.78/0.89 | 0.67/0.86 |
|  |  |  |  |  |
| T2 | STEE | 0.67/0.35 | 1.00/0.55 | 0.80/0.43 |
|  | HGG-G4 | 0.75/0.60 | 0.50/0.33 | 0.60/0.43 |
|  | HGG-G3 | 0.78/0.90 | 0.78/0.96 | 0.78/0.93 |
|  |  |  |  |  |
| ADC | STEE | 0.00/0.33 | 0.00/0.27 | 0.00/0.30 |
|  | HGG-G4 | 0.50/0.65 | 0.50/0.61 | 0.50/0.63 |
|  | HGG-G3 | 0.58/0.83 | 0.78/0.93 | 0.67/0.88 |
|  |  |  |  |  |
